# Biomarker results from a phase II study of MEK1/2 inhibitor binimetinib (MEK162) in patients with advanced *NRAS*- or *BRAF*-mutated melanoma

**DOI:** 10.18632/oncotarget.26753

**Published:** 2019-03-05

**Authors:** Carla M.L. van Herpen, Sanjiv S. Agarwala, Axel Hauschild, Carola Berking, J. Thaddeus Beck, Dirk Schadendorf, Rob Jansen, Paola Queirolo, Paolo A. Ascierto, Christian U. Blank, Michael C. Heinrich, Rupam R. Pal, Adnan Derti, Victor Antona, Heidi Nauwelaerts, Angela Zubel, Reinhard Dummer

**Affiliations:** ^1^ Department of Medical Oncology, Radboud University Medical Center, Nijmegen, The Netherlands; ^2^ Department of Medical Oncology and Hematology, St. Luke's University Health Network, Bethlehem, PA, USA; ^3^ Department of Dermatology, University Hospital Schleswig-Holstein, Kiel, Germany; ^4^ Department of Dermatology, University Hospital of Munich (LMU), Munich, Germany; ^5^ Department of Oncology, Highlands Oncology Group, Fayetteville, AR, USA; ^6^ Department of Dermatology, University Hospital Essen, Essen, Germany; ^7^ Department of Medical Oncology, Maastricht University Medical Center, Maastricht, The Netherlands; ^8^ Department of Medical Oncology, IRCCS San Martino, IST Istituto Nazionale per la Ricerca sul Cancro, Genoa, Italy; ^9^ Unit of Melanoma, Cancer Immunotherapy and Innovative Therapy, Istituto Nazionale Tumori Fondazione Pascale, Naples, Italy; ^10^ Department of Medical Oncology, The Netherlands Cancer Institute, Amsterdam, The Netherlands; ^11^ Department of Medicine, Veterans Administration Portland Health Care System and Oregon Health and Science University Knight Cancer Institute, Portland, OR, USA,; ^12^ Biostatistics, Novartis Healthcare Private Limited, Hyderabad, India; ^13^ Department of Translational Oncology, Novartis Institutes for BioMedical Research, Cambridge, MA, USA; ^14^ Department of Translational Oncology, Novartis Institutes for BioMedical Research, Basel, Switzerland; ^15^ Department of Dermatology, University Hospital Zurich, Zurich, Switzerland

**Keywords:** binimetinib, MEK inhibitor, biomarker, melanoma, phase II

## Abstract

*BRAF* and *RAS* are the most frequently mutated mitogen-activated protein kinase (MAPK) genes in melanoma. Binimetinib is a highly selective MAPK kinase (MEK) 1/2 inhibitor with clinical antitumor activity in *NRAS*- and *BRAF*^V600^-mutant melanoma. We performed a nonrandomized, open-label phase II study, where 183 metastatic melanoma patients received binimetinib 45 mg / 60 mg twice-daily (*BRAF* arms), or binimetinib 45 mg twice-daily (*NRAS* arm). Biomarker analyses were prespecified as secondary and exploratory objectives. Here we report the extent of MAPK pathway inhibition by binimetinib, genetic pathway alterations of interest, and potential predictive markers for binimetinib efficacy. Twenty-five fresh pre- and post-dose tumor sample pairs were collected for biomarker analyses, which included assessment of binimetinib on MEK/MAPK signaling by pharmacodynamic analysis of pERK and DUSP6 expression in pre- vs post-dose tumor biopsies; identification of pERK and DUSP6 expression/efficacy correlations; assessment of baseline tumor molecular status; and exploration of potential predictive biomarkers of efficacy of binimetinib. The postbaseline pERK and DUSP6 expression decreased across all arms; no association between reduced pERK or DUSP6 levels with clinical efficacy was observed. Genetic aberrations were similar to previously reported data on clinical melanoma samples. Genetic pathway alterations occurred predominantly within *CDKN2A/B*, *PTEN*, and *TRRAP* (*BRAF*-mutation) and *CDKN2A/B*, *TP53*, and *NOTCH2* (*NRAS*-mutation). Several patients with BRAF mutations had amplification of genes on chromosome 7q; these patients tended to have shorter progression-free survival than other patients with *BRAF*-mutant melanoma. Further analysis of genetic alterations, including amplifications of growth factor genes, will determine utility as biomarkers for efficacy.

## INTRODUCTION

The MAPK signaling pathway (i.e., RAS-RAF-MEK-ERK pathway) regulates cellular proliferation, survival, and differentiation and contributes to the pathogenesis of melanoma [1]. Multiple genetic changes can lead to hyperactivation of this pathway, and are involved in the pathogenesis of various solid tumor types, including melanoma and thyroid, colorectal, and ovarian cancer [2–5]. Constitutive MAPK pathway activation in cancer can occur through several mechanisms, most frequently via mutations in *BRAF* or *RAS*. Activating *NRAS* and *BRAF* mutations are present in approximately 20% [6, 7] and 50% [6, 8] of primary cutaneous melanomas, respectively.

A number of therapies that directly target the MAPK pathway have been approved for *BRAF*-mutated melanoma, including the BRAF^V600^- specific inhibitors vemurafenib (single agent), and dabrafenib (single agent or in combination with trametinib), and mitogen-activated protein kinase (MEK) inhibitors trametinib (single agent or in combination with dabrafenib) and cobimetinib (in combination with vemurafenib) [9–15]. The combination of BRAF and MEK inhibitor therapies has shown significantly improved benefit over BRAF inhibitor monotherapy in phase III clinical trials [16–18]. In addition to the nonspecific immunotherapies, three immune checkpoint modulators, ipilimumab (anti-cytotoxic T-lymphocyte associated antigen 4 [CTLA-4]), nivolumab (anti–programmed cell death protein 1 [PD-1]), and pembrolizumab (anti–PD-1), have been approved for the treatment of melanoma [19].

Therapies targeting either mutated *BRAF* or *NRAS* alone can encounter a number of challenges, including resistance to BRAF inhibitors, which occurs frequently, mostly through reactivation of the MAPK pathway; common paths to resistance include *BRAF* amplification or alternative splicing and mutations in *RAS*, *MAP2K1*, and *CDKN2A* [20, 21], among other mechanisms.

In contrast to *BRAF*-mutated melanoma, no approved targeted therapies exist for *NRAS*-mutated melanoma, but positive phase III data on PFS have been reported for a study comparing the efficacy of binimetinib (MEK162) versus dacarbazine in unresectable or metastatic *NRAS*-mutated melanoma (NEMO study) [22]. Binimetinib is an oral, selective, ATP-uncompetitive inhibitor of MEK 1 and MEK 2 [23].

Binimetinib has shown promising results in *BRAF*-mutated melanoma, both alone [24] and in combination [25]. In addition, preclinical MEK inhibitor activity has been shown in *BRAF*-mutated melanoma [26]. The safety profile of binimetinib and preliminary signs of antitumor activity were shown in a phase I trial in patients with advanced solid tumors [27, 28]. This open-label phase II study assessed the use of binimetinib in patients with *BRAF*^V600^- or *NRAS*-mutated advanced melanoma. The efficacy and safety results from an earlier data cut-off of February 29, 2012 (smaller subgroups of patients with *NRAS*- or *BRAF*-mutated melanoma) have been previously reported [24]. No patients had a complete response, and 6 of 30 patients (20%) with *NRAS*-mutated melanoma (3 confirmed) and 8 of 41 patients (20%) with *BRAF*-mutated melanoma (2 confirmed) had a partial response. Binimetinib was the first targeted therapy to show activity in patients with *NRAS*-mutated melanoma.

In this study (NCT01320085), biomarker data from binimetinib-treated patients with *NRAS*- and *BRAF*-mutated melanoma were analysed as prespecified secondary and exploratory objectives to investigate the extent of MAPK pathway inhibition and further genetic pathway alterations, in order to find potential predictive markers of response to binimetinib. Among others, dual-specificity phosphatase 6 (DUSP6) and phosphorylated extracellular signal-regulated kinase (pERK) are known/predicted biomarkers of MAPK inhibition and could potentially predict the extent of response to treatment with binimetinib [20, 29].

## RESULTS

### Patient disposition and characteristics

At the trial data cut-off date (7 January 2014), a total of 183 patients were enrolled. Sixty-six patients with *BRAF* mutations were treated: 41 received binimetinib 45 mg twice daily (BID), and 25 received binimetinib 60 mg BID (subsequently reduced to 45 mg BID). A total of 117 patients with *NRAS* mutations received binimetinib 45 mg BID (Table 1). Patient demographics and disease characteristics are shown in Supplementary Table 1.

**Table 1:** Patient disposition

	BRAF-mutant		*NRAS-*mutant	All patients
	Binimetinib 45 mg (*n* = 41)	Binimetinib 60 mg (*n* = 25)	Binimetinib 45 mg (*n* = 117)	*N* = 183
Patients treated, *n* (%)				
Treatment discontinued	41 (100)	23 (92.0)	104 (88.9)	168 (91.8)
Treatment ongoing^a^	0	2 (8.0)	13 (11.1)	15 (8.2)
Primary reason for end of treatment, *n* (%)				
Adverse event(s)^b^	12 (29.3)	5 (20.0)	14 (12.0)	31 (16.9)
Patient withdrew consent	2 (4.9)	1 (4.0)	4 (3.4)	7 (3.8)
Disease progression	26 (63.4)	16 (64.0)	86 (73.5)	128 (69.9)
Protocol deviation	1 (2.4)	1 (4.0)	0	2 (1.1)
Duration of exposure, median (range), weeks	9.6 (1.1–26.6)	8.0 (2.0–102)	15.9 (0.3–87.9)	11.6 (0.3–102.0)
*BRAF* mutation status, *n* (%)^c^				
None (no wild-type mutation detected)	0	0	3 (2.6)	
V600E	34 (82.9)	19 (76.0)	0	
V600K	5 (12.2)	1 (4.0)	0	–
Unknown mutation	1 (2.4)^f^	2 (8.0)^f^	0	
Other (mutations other than V600E/K)^d^	1 (2.4)^g^	0	0	
Missing (no V600 *BRAF* mutation data)^e^	0	3 (12.0)^f^	114 (97.4)	
*NRAS* mutation status, *n* (%)^d^				
None (no mutation detected)	0	5 (20.0)	4 (3.4)^h^	
Q61	0	0	100 (85.5)	
G12/13	0	0	2 (1.7)	–
Unknown mutation^c^	1 (2.4)	0	1 (0.9)^i^	
Missing (no *NRAS* mutation data)	40 (97.6)	20 (80.0)	10 (8.5)^j^	
Clinical activity^k^ [24]	(*n* = 35)	NR	(*n* = 28)	
DCR, *n* (%)	21 (60)	NR	19 (68)	

^a^Treatment ongoing at the time of the cut-off (Jan 7, 2014)

^b^Includes fatal case with liver failure in the BRAF-mutated 60-mg treatment group;

^c^Any other mutation not known; known mutation includes all Q61, A59T, A11T, G12V, G13R;

^d^Any other known mutation (L597, D594, G606, K60);

^e^Analysis results are missing;

^f^V600E according to local laboratory;

^g^V600R according to local laboratory;

^h^Three Q61R (1 result received after database lock) and one G12 (result received after database lock);

^i^Q61R according to local laboratory;

^j^Three Q61R, two Q61L, two Q61K, two Q61, and one G12 mutation according to local laboratory.

^k^Data for clinical activity available for 63 patients for response-rate analysis set (Ascierto, 2013).

DCR, disease control rate; NR, not reported.

### Efficacy and safety analysis

Efficacy and safety results for this study have been previously reported for the *BRAF*- and *NRAS-*mutated arms for two data cut-off points, 29 February 2012 [24] and 7 January 2014 [31]. Biomarker results presented herein are derived from the later cut-off date.

### Biomarker analysis

Biomarkers were analyzed to evaluate MAPK pathway inhibition and analysis was undertaken to evaluate on-treatment biomarker expression and frequency of tumor genetic alterations at baseline. Results were compared against melanoma cases in The Cancer Genome Atlas (TCGA) database and in the context of clinical outcomes, where appropriate. Twenty-five fresh, paired (baseline and on Cycle 1, Day 15) tumor samples were collected for pharmacodynamic biomarker analysis. Fifteen pairs were evaluable for pERK analysis (three pairs in the *BRAF-*mutated 45 mg subgroup; four and eight pairs, respectively, in the *BRAF*-mutated 60 mg and *NRAS*-mutated arms). Fourteen pairs were evaluable for DUSP6 analysis (three pairs each in the *BRAF-*mutated 45 mg and 60 mg arms and eight pairs in the *NRAS*-mutated arm).

**Figure 1:**
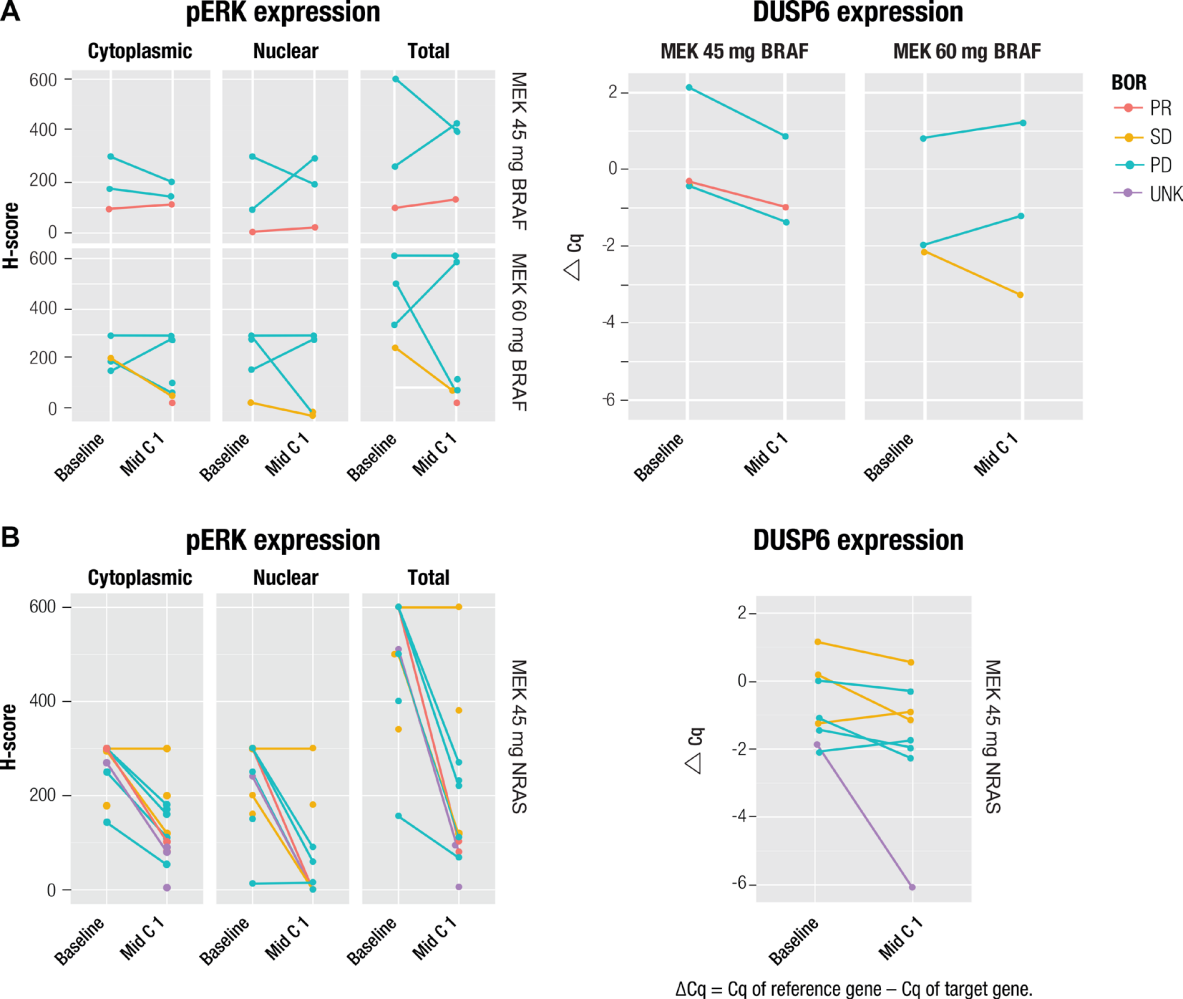
Change from baseline in pERK and DUSP6 expression in patients with (**A)** *BRAF* or **(B)** *NRAS* mutation and correlation with best overall response. Single dots represent unpaired biopsies. BOR, best overall response; Cq, quantification cycle; PD, progressive disease; PR, partial response; SD, stable disease; UNK, unknown.

Pharmacodynamic analysis of postbaseline pERK and DUSP6 expression in patients with *BRAF* and *NRAS* mutations showed MAPK pathway inhibition (Figure 1). Decreased postbaseline cytoplasmic and nuclear pERK expression was observed in 11 of 15 and in 9 of 15 paired samples, respectively, and decreased total DUSP6 expression postbaseline was observed in 10 of 14 paired samples. Median reduction in pERK H-score was 47% and 70% in the cytoplasmic and nuclear compartments, respectively, and median DUSP6 reduction, in Δ Ct, was 36%. MAPK pathway inhibition was shown in both responders and nonresponders, with no apparent association between reduced expression of either pERK or DUSP6 with overall response rates.

Comparison of patients in the *BRAF*- and *NRAS*-mutated arms with TCGA melanoma cases showed overall concordance of the tumor genetic landscape with regard to the percentage of patients experiencing alterations in the most frequently mutated genes (Supplementary Figure 1A and 1B). Within the *BRAF*-mutated group, concordance was observed in *PTEN*, *TRRAP*, and *TP53*. Slightly more *CDKN2A* alterations and fewer *CDKN2B* alterations were observed in study patients compared with the cases in the TCGA database, possibly due to higher sequencing depth and more systematic annotation of variants in this study, respectively. Within the *NRAS*-mutated group, concordance was observed in *CDKN2A/B*, *TP53*, and *NOTCH2*. There were no trends showing any association between efficacy and the subtype of *NRAS Q61* mutations (Supplementary Figure 2). Supplementary Table 2 provides additional context for the BRAF and NRAS mutations, including mutation type and presence or absence in the Catalogue of Somatic Mutations in Cancer (Supplementary Table 2).

**Figure 2:**
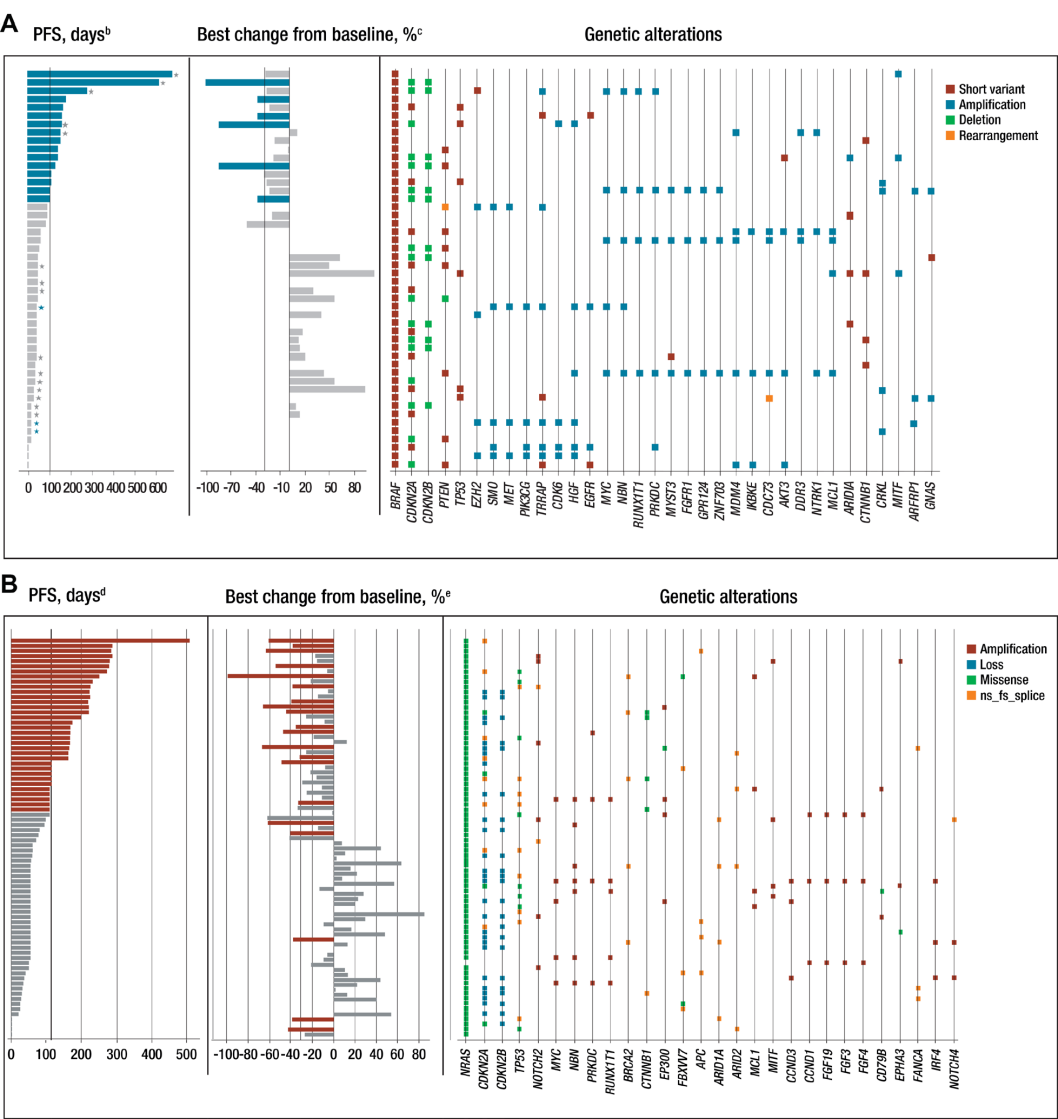
Genetic landscape of tumor samples and efficacy in patients with (**A**) *BRAF* mutation^a^ and (**B**) *NRAS* mutation. ^a^The genetic landscape shows gene alterations that occurred in ≥ 3 patients in the *BRAF*-mutant population; ^b^Blue indicates patients with PFS ≥ 3.5 months (median PFS in the 45 mg BRAF^V600^ arm); ^c^Blue indicates patients with reduction in sum of the longest diameter from baseline ≥ 30%; ^d^Red indicates patients with PFS ≥ 3.6 months (median PFS in the *NRAS*-mutated arm); ^e^Red indicates patients with reduction in sum of the longest diameter from baseline ≥ 30%; ^*^Patients in the *BRAF*-mutant group receiving binimetinib 60 mg BID who were dose reduced to 45 mg BID; ^*^Patients in the *BRAF*-mutant group receiving binimetinib 60 mg BID who were not dose reduced. *MET* and *HGF* amplifications in the *BRAF* panel *CCND3* and *CCND1* amplifications in the *NRAS* panel are indicated with a black box. BID, twice daily; fs, frameshift; ns, nonsense; PFS, progression-free survival.

A weak association between specific mutations or total number of mutations with either measure of efficacy was shown among patients with either *BRAF*- or *NRAS*-mutated melanoma (Figure 2A and 2B, respectively). Notable differences between *BRAF*- and *NRAS*-mutated melanomas at baseline included *PTEN* (21% vs 2.5% of patients, respectively) and *P53* (13% vs 22%, respectively). Five *BRAF*-mutated tumors had broad amplifications on chromosome 7 and five others on chromosome 1, while three *NRAS*-mutated tumors exhibited amplifications on chromosome 11. Amplifications tended to be associated with shorter progression-free survival (PFS); for example, amplifications in *CCND1* or *CCND3* occurred only in five *NRAS*-mutated patients with a PFS shorter than the median (≤ 3.6 months).

Beyond individual genes, pathways or coherent classes of genes were examined, namely the cell cycle and PI3K and P53 pathways as well as epigenetic regulators, transcription factors, and DNA damage response genes. The rationale for these groupings is that genes with parallel or related functions relevant to melanoma may harbor mutually exclusive mutations, as is the case for *BRAF* and *NRAS*, such that they would escape notice when examined individually. Among both *BRAF*- and *NRAS*-mutated arms, genetic pathway alterations were observed, predominantly within the cell cycle genes (Figure 2A and 2B; Supplementary Table 3). Within the *BRAF*-mutated group, these alterations were driven primarily by *CDKN2A*, which was altered in 27 of 48 patients (56.3%). Among the *NRAS*-mutated group, 45 of 78 patients (58%) had alterations in the cell cycle genes; 27 of 65 patients (42%) had both alterations within the cell cycle genes and a short PFS (≤ 110 days).

## DISCUSSION

The biomarker analyses of this study aimed to describe the biological impact of MEK-targeted inhibition of the MAPK pathway with binimetinib, establish the *BRAF*- and *NRAS*-mutated tumor genetic landscape, and explore the link between genetic pathway alterations and the response to binimetinib. Understanding the biological impact of MEK inhibition, and the predictive value of tumor genetic markers and pathway alterations on response, could be invaluable for optimizing the efficacy of targeted therapy. Pharmacodynamic analyses showed MAPK pathway inhibition by binimetinib on Day 15 through decreased pERK levels and DUSP6 gene expression. These decreases were observed in both *BRAF-* and *NRAS*-mutated arms, and were moderate and consistent with observations in other cancer studies with MEK inhibitors, such as the observed suppression of pERK by cobimetinib (GDC-0973) both *in vitro* and *in vivo* [32]. However, no association between reduced pERK or DUSP6 levels with clinical efficacy was observed, likely due to limited data or sampling time points. Another consideration is that in certain genomic contexts, including some tumors with RAS mutations (ie, preclinical data of BRAF/RAS-WT tumor cells) [33], the MEK-ERK pathway may not be fundamental for tumor cell proliferation; thus, inhibition of the MEK-ERK pathway may not reduce the survival of certain tumor cells.

Overall, the tumor genetic landscape for patients with *BRAF*- and *NRAS*-mutated melanoma was concordant with that reported in TCGA melanoma cases. This observation demonstrated a consistency of genetic alterations in this patient subset with historical samples. This equivalence also contributes to the validation of these biomarker results, leading toward a better understanding of changes that occur within patients with *BRAF*- and *NRAS*-mutated melanoma and their predictive value.

Several patients with *BRAF*-mutated tumors had amplification of genes on the long arm of chromosome 7 (7q); seven patients exhibited *MET* and/or *HGF* amplifications, with coamplification occurring in three of them. Five of these seven patients (including the three patients with coamplification) had a PFS that was shorter than the median PFS of 3.5 months for the population with *BRAF* mutations. Particular genes of high interest on 7q include *HGF*, *MET*, *EZH2*, and *SMO*. The latter two have been associated with driving the progression of melanoma [34, 35]; *HGF* and *MET* form a functional pair since they are cognate ligand and receptor, respectively. Among patients in the *NRAS*-mutated group, *CCND1* or *CCND3* amplifications were exclusively seen in five patients with shorter PFS, indicating that constitutive *CDK4/6* pathway signaling may lead to resistance. In this regard, a phase Ib/II study (NCT01781572) with binimetinib in combination with the CDK4/6 inhibitor ribociclib (LEE011) in patients with *NRAS*-mutant melanoma has recently completed, with preliminary data from the initial Phase 1b study suggesting a manageable safety profile and favourable efficacy [36]. Furthermore, positive phase III data have been reported for the NEMO study comparing the efficacy of binimetinib single agent versus dacarbazine in unresectable or metastatic *NRAS*-mutant melanoma [22]. We cannot rule out other genetic alterations that were observed in patients with progressive disease that may have had an effect on PFS.

Binimetinib showed activity in patients with *BRAF-* or *NRAS-*mutated melanoma through MAPK pathway inhibition, and genomic profiling highlighted genetic alterations of interest that could be used as potential predictive biomarkers of response to binimetinib. Although all comparisons with patient outcomes for these data are currently observational in nature, they are indicative of the potential predictive use of genetic data in future larger cohorts. Combined with interpretation of biomarker data from other ongoing studies of binimetinib, these data could provide context toward understanding the biological impact of and prediction of response to binimetinib. Of relevance, assessment of potential additional biomarkers of efficacy or safety was incorporated in the aforementioned NEMO trial, in the phase III COLUMBUS trial (NCT01909453) comparing binimetinib plus encorafenib with encorafenib or vemurafenib in patients with *BRAF*-mutated melanoma, and in the phase II LOGIC-2 trial (NCT02159066) investigating sequential encorafenib/binimetinib combination therapy followed by a combination with targeted agents after disease progression in patients with *BRAF*^V600^ -mutated melanoma.

## MATERIALS AND METHODS

### Patients

Details of the study have been published previously [24]. Briefly, baseline *BRAF* or *NRAS* status was assessed using archival or fresh tumor biopsies either at a local or central laboratory (MolecularMD) and analyzed by a semiquantitative polymerase chain reaction (*BRAF*) or bidirectional Sanger sequencing assay (*NRAS*). After patient enrollment, all tumor biopsies assessed at local laboratories were sent to the central laboratory for mutational status confirmation.

The study was designed, undertaken, and reported in accordance with the Declaration of Helsinki and the ICH Harmonised Tripartite Guideline for Good Clinical Practice. The protocol was approved by an institutional review board, independent ethics committee, or research ethics board at each institution. All patients provided written informed consent before screening and additional consent if participating in the exploratory biomarker analysis.

### Study design and treatments

This study was a nonrandomized, open-label phase II study in which patients were divided into one of three treatment arms according to tumor *NRAS* or *BRAF* status: binimetinib 45 mg BID or 60 mg BID for *BRAF*-mutant tumors, or binimetinib 45 mg BID for *NRAS*-mutant tumors. The 60 mg BID dose of binimetinib for patients with *BRAF*-mutated tumors was subsequently reduced to 45 mg BID per a protocol amendment following two serious adverse events (grade 4 acute liver failure in 1 patient; grade 3 cardiomyopathy, decreased ejection fraction and tachycardia in a second patient). Treatment was administered in 28-day cycles. Binimetinib was administered orally (film-coated tablet) BID from Day 1 of Cycle 1 and continuously throughout the study.

The primary endpoint was the proportion of patients who achieved an objective response (complete response + partial response). Secondary endpoints included PFS, time to response, safety, tolerability, pharmacokinetics, and pharmacodynamics. Biomarker analyses, the focus of this manuscript, were prespecified secondary and exploratory objectives, and included: assessment of pharmacodynamic effects of binimetinib on MEK/MAPK signaling by analysis of pERK and DUSP6 gene expression in pre- versus post-dose tumor biopsies; examination of correlations between pERK, DUSP6 expression and efficacy; assessment of the baseline molecular status of the tumors and exploration of potential predictive biomarkers of response to binimetinib.

### Study procedures

Safety and pharmacokinetic assessments and overall efficacy data from an earlier cut-off were reported previously [24]. Biomarker-related efficacy data are presented here. A whole blood sample (∼6.0 mL) was taken from all patients (at Cycle 1 Day 1) to provide a non-tumorous tissue sample to perform genetic analysis (if compliant with local IRB requirements) This sample was analyzed to compare tumor-specific gene alterations in DNA from tumor samples with DNA from normal-non-tumor cells. Baseline and on-study (Cycle 1, Day 15) fresh tumor biopsy samples were collected from patients in all three treatment arms and analyzed for the pharmacodynamic markers pERK and DUSP6. Immunohistochemistry (IHC) data reported from the lab included quantitative data (eg, percent tumor and percent positive cells) or a semi quantitative measure of protein expression reported as 3 individual components, 1+ 2+ and 3+. The pathologist determined whether the staining in a cellular compartment was absent (0+), slight (1+), moderate (2+), or strong (3+). The H-Score used to assess pERK for each cellular compartment was then calculated as the sum of (the percentages of stained cells * their intensity), or (%1+) + (2 * %2+) + (3 * %3+) and ranged between 0 and 300. Δ Ct (cycle threshold), a relative measure of the concentration of target in the PCR reaction, was used to measure DUSP-6. Δ Ct is the normalization of Raw Ct that is calculated by subtracting the baseline (reference sample): (Δ Ct = Ct Gene of interest – Ct Internal control). Deep sequencing of formalin-fixed, paraffin-embedded tumor samples from enrolled patients was used to profile genomic alterations in 296 cancer-related genes in order to identify potential predictive markers of binimetinib sensitivity, as previously described [30]. Briefly, DNA was sequenced at high depth (median 744X) on an Illumina HiSeq 2500 sequencer following probe-based targeted exome capture.

Analyses were descriptive and exploratory in nature, and no inferential analysis was performed. Data were summarized with respect to demographic and baseline characteristics and all relevant pharmacodynamics and genetic alteration measurements.

## SUPPLEMENTARY MATERIALS FIGURES AND TABLES



## References

[1] Vennepureddy A, Thumallapally N, Motilal Nehru V, Atallah JP, Terjanian T (2016). Novel drugs and combination therapies for the treatment of metastatic melanoma. J Clin Med Res.

[2] Frémin C, Meloche S (2010). From basic research to clinical development of MEK1/2 inhibitors for cancer therapy. J Hematol Oncol.

[3] Konieczkowski DJ, Johannessen CM, Abudayyeh O, Kim JW, Cooper ZA, Piris A, Frederick DT, Barzily-Rokni M, Straussman R, Haq R, Fisher DE, Mesirov JP, Hahn WC (2014). A melanoma cell state distinction influences sensitivity to MAPK pathway inhibitors. Cancer Discov.

[4] Pratilas CA, Solit DB (2010). Targeting the mitogen-activated protein kinase pathway: physiological feedback and drug response. Clin Cancer Res.

[5] Chappell WH, Steelman LS, Long JM, Kempf RC, Abrams SL, Franklin RA, Bäsecke J, Stivala F, Donia M, Fagone P, Malaponte G, Mazzarino MC, Nicoletti F (2011). Ras/Raf/MEK/ERK and PI3K/PTEN/Akt/mTOR inhibitors: rationale and importance to inhibiting these pathways in human health. Oncotarget.

[6] Jakob JA, Bassett RL, Ng CS, Curry JL, Joseph RW, Alvarado GC, Rohlfs ML, Richard J, Gershenwald JE, Kim KB, Lazar AJ, Hwu P, Davies MA (2012). NRAS mutation status is an independent prognostic factor in metastatic melanoma. Cancer.

[7] Lee JH, Choi JW, Kim YS (2011). Frequencies of BRAF and NRAS mutations are different in histological types and sites of origin of cutaneous melanoma: A meta-analysis. Br J Dermatol.

[8] Colombino M, Capone M, Lissia A, Cossu A, Rubino C, De Giorgi V, Massi D, Fonsatti E, Staibano S, Nappi O, Pagani E, Casula M, Manca A (2012). BRAF/NRAS mutation frequencies among primary tumors and metastases in patients with melanoma. J Clin Oncol.

[9] Zelboraf (vemurafenib) (2017). Prescribing Information.

[10] Zelboraf (vemurafenib) (2018). Summary of Product Characteristics.

[11] Tafinlar (dabrafenib) (2018). Prescribing Information.

[12] Tafinlar (dabrafenib) (2018). Summary of Product Characteristics.

[13] Mekinist (trametinib) (2018). Prescribing Information.

[14] Mekinist (trametinib) (2018). Summary of Product Characteristics.

[15] Cotellic (cobimetinib) (2018). Prescribing Information.

[16] Long GV, Stroyakovskiy D, Gogas H, Levchenko E, de Braud F, Larkin J, Garbe C, Jouary T, Hauschild A, Grob JJ, Chiarion Sileni V, Lebbe C, Mandalà M (2014). Combined BRAF and MEK inhibition versus BRAF inhibition alone in melanoma. N Engl J Med.

[17] Robert C, Karaszewska B, Schachter J, Rutkowski P, Mackiewicz A, Stroiakovski D, Lichinitser M, Dummer R, Grange F, Mortier L, Chiarion-Sileni V, Drucis K, Krajsova I (2015). Improved overall survival in melanoma with combined dabrafenib and trametinib. N Engl J Med.

[18] Larkin J, Ascierto PA, Dréno B, Atkinson V, Liszkay G, Maio M, Mandalà M, Demidov L, Stroyakovskiy D, Thomas L, de la Cruz-Merino L, Dutriaux C, Garbe C (2014). Combined vemurafenib and cobimetinib in BRAF-mutated melanoma. N Engl J Med.

[19] Dillon AB, Lin K, Kwong A, Ortiz S (2015). Immunotherapy in melanoma, gastrointestinal (GI), and pulmonary malignancies. AIMS Public Health.

[20] Shi H, Hugo W, Kong X, Hong A, Koya RC, Moriceau G, Chodon T, Guo R, Johnson DB, Dahlman KB, Kelley MC, Kefford RF, Chmielowski B (2014). Acquired resistance and clonal evolution in melanoma during BRAF inhibitor therapy. Cancer Discov.

[21] Van Allen EM, Wagle N, Sucker A, Treacy DJ, Johannessen CM, Goetz EM, Place CS, Taylor-Weiner A, Whittaker S, Kryukov GV, Hodis E, Rosenberg M, McKenna A (2014). The genetic landscape of clinical resistance to RAF inhibition in metastatic melanoma. Cancer Discov.

[22] Dummer R, Schadendorf D, Ascierto PA, Arance A, Dutriaux C, Di Giacomo AM, Rutkowski P, Del Vecchio M, Gutzmer R, Mandala M, Thomas L, Demidov L, Garbe C (2017). Binimetinib versus dacarbazine in patients with advanced NRAS-mutant melanoma (NEMO): a multicentre, open-label, randomised, phase 3 trial. Lancet Oncol.

[23] Lee PA, Wallace E, Marlow A, Yeh T, Marsh V, Anderson D, Woessner R, Hurley B, Lyssikatos J, Poch G, Gross S, Rana S, Winski S, Koch K (2010). Preclini cal development of ARRY-162, a potent and selective MEK 1/2 inhibitor. Cancer Res.

[24] Ascierto PA, Schadendorf D, Berking C, Agarwala SS, van Herpen CM, Queirolo P, Blank CU, Hauschild A, Beck JT, St-Pierre A, Niazi F, Wandel S, Peters M (2013). MEK162 for patients with advanced melanoma harbouring NRAS or Val600 BRAF mutations: a non-randomised, open-label phase 2 study. Lancet Oncol.

[25] van Herpen C, Postow M, Carlino M, Kalkavan H, Weise A, Amaria RN, De Vos F, Carvajal RD, Matano A, Bhansali S, Lam L, Yerramilli-Rao P, Sosman JA (2015). A phase 1b/2 study of ribociclib (LEE011; CDK4/6 inhibitor) in combination with binimetinib (MEK162; MEK inhibitor) in patients with *NRAS*-mutant melanoma. Eur J Cancer.

[26] Winski S, Anderson D, Bouhana K, Impastato R, Woessner R, Zuzack J, Tunquist B, Garrus J, Pheneger T, Lee P (2010). MEK162 (ARRY-162), a novel MEK 1/2 inhibitor, inhibits tumor growth regardless of KRas/Raf pathway mutations. Eur J Cancer.

[27] Bendell JC, Papadopoulos K, Jones SF, Barrett E, Guthrie K, Kass CL, Litwiler KS, Napier C, Patnaik A (2011). Abstract B243: A phase I dose-escalation study of MEK inhibitor MEK162 (ARRY-438162) in patients with advanced solid tumors. Mol Cancer Ther.

[28] Finn R, Javle M, Tan B, Weekes C, Bendell J, Patnaik A, Khan G, Laheru D, Anderson L, Christy-Bittel J, Barrett E, Guthrie K, Litwiler K, Bekaii-Saab TS (2012). A phase I study of MEK inhibitor MEK162 (ARRY-438162) in patients with biliary tract cancer. J Clin Oncol.

[29] Li W, Song L, Ritchie AM, Melton DW (2012). Increased levels of DUSP6 phosphatase stimulate tumourigenesis in a molecularly distinct melanoma subtype. Pigment Cell Melanoma Res.

[30] Frampton G, Fichtenholtz A, Otto GA, Wang K, Downing SR, He J, Schnall-Levin M, White J, Sanford EM, An P, Sun J, Juhn F, Brennan K (2013). Development and validation of a clinical cancer genomic profiling test based on massively parallel DNA sequencing. Nat Biotechnol.

[31] van Herpen C, Agarwala SS, Hauschild A, Dummer R, Berking C, Beck JT, Schadendorf D, Gibney GT, Jansen R, Queirolo P, Ascierto PA, Blank CU, Nauwelaerts H (2014). Overall survival and biomarker results from a phase 2 study of MEK1/2 inhibitor binimetinib (MEK162) in patients with advanced NRAS-mutant melanoma. Ann Oncol.

[32] Hoeflich KP, Merchant M, Orr C, Chan J, Den Otter D, Berry L, Kasman I, Koeppen H, Rice K, Yang NY, Engst S, Johnston S, Friedman LS (2012). Intermittent administration of MEK inhibitor GDC-0973 plus PI3K inhibitor GDC-0941 triggers robust apoptosis and tumor growth inhibition. Cancer Res.

[33] Solit DB, Garraway LA, Pratilas CA, Sawai A, Getz G, Basso A, Ye Q, Lobo JM, She Y, Osman I, Golub TR, Sebolt-Leopold J, Sellers WR (2006). BRAF mutation predicts sensitivity to MEK inhibition. Nature.

[34] Zingg D, Debbache J, Schaefer SM, Tuncer E, Frommel SC, Cheng P, Arenas-Ramirez N, Haeusel J, Zhang Y, Bonalli M, McCabe MT, Creasy CL, Levesque MP (2015). The epigenetic modifier EZH2 controls melanoma growth and metastasis through silencing of distinct tumour suppressors. Nat Commun.

[35] Santini R, Vinci MC, Pandolfi S, Penachioni JY, Montagnani V, Olivito B, Gattai R, Pimpinelli N, Gerlini G, Borgognoni L, Stecca B (2012). Hedgehog-GLI signaling drives self-renewal and tumorigenicity of human melanoma-initiating cells. Stem Cells.

[36] Schuler MH, Ascierto PA, Leon De Vos FVF, Postow MA, Van Herpen CML, Carline MS, Sosman JA, Berking C, Long GV, Weise A, Gutzmer R, Kaatz M (2017). Phase 1b/2 trial of ribociclib+binimetinib in metastatic *NRAS*-mutant melanoma: Safety, efficacy, and recommended phase 2 dose (RP2D). J Clin Oncol.

