# Supplementary material for: Biomarker results from a phase II study of MEK1/2 inhibitor binimetinib (MEK162) in patients with advanced NRAS- or BRAF-mutated melanoma
Source: Oncotarget. 2019 Mar 5;10(19):1850–9. doi: 10.18632/oncotarget.26753 (PMC6442999; doi:10.18632/oncotarget.26753)
Supplement: Supplementary file 1 [file oncotarget-10-1850-s001.pdf]

Biomarker results from a phase II study of MEK1/2 inhibitor binimetinib (MEK162) in patients with advanced *NRAS*- or *BRAF*-mutated melanoma

SUPPLEMENTARY MATERIALS

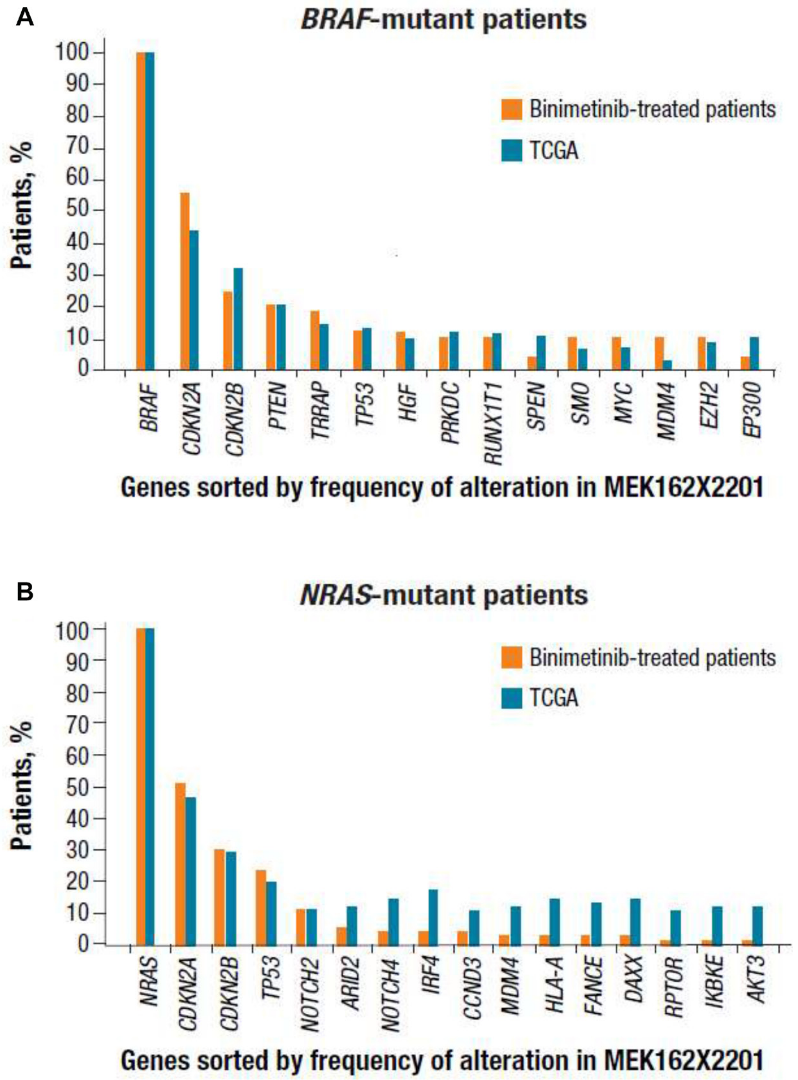

Supplementary Figure 1: Study data on gene alterations in (**A**) *BRAF*- and (**B**) *NRAS*-mutated melanoma patients treated with binimetinib vs data from The Cancer Genome Atlas (TCGA). Genes sorted by frequency of alteration in binimetinib-treated patients. TCGA, The Cancer Genome Atlas.

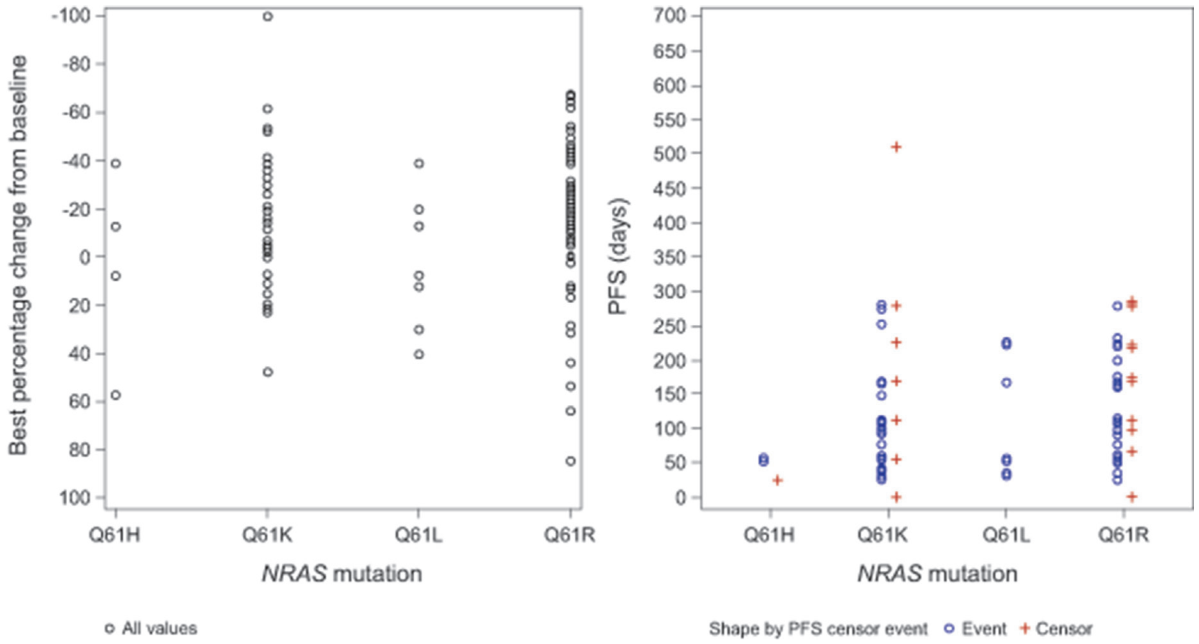

Supplementary Figure 2: Best percentage change from baseline in sum of the longest diameter and progression-free survival by different Q61 mutation types for *NRAS*-mutated patients. PFS, progression-free survival.

Supplementary Table 1: Patient demographics and baseline characteristics

	***BRAF*-mutated patients receiving binimetinib 45 mg (*n* = 41)**	***BRAF*-mutated patients receiving binimetinib 60 mg (*n* = 25)**	***NRAS*-mutated patients receiving binimetinib 45 mg (*n* = 117)**	**All patients (*N* = 183)**
Age, median (range), years	57 (21–81)	52 (27–70)	62 (26–84)	58 (21–84)
Sex, *n* (%)				
Male/Female	22 (53.7)/19 (46.3)	8 (32.0)/17 (68.0)	84 (71.8)/33 (28.2)	114 (62.3)/69 (37.7)
WHO performance status, *n* (%)				
0/1/2	33 (80.5)/8 (19.5)/0	19 (76.0)/5 (20.0)/1 (4.0)	82 (70.1)/30 (25.6)/5 (4.3)	134 (73.2)/43 (32.5)/6 (3.3)
Melanoma type, *n* (%)				
Cutaneous	40 (97.6)	23 (92.0)	111 (94.9)	174 (95.1)
Noncutaneous	0	0	4 (3.4)[a]	4 (2.2)
Missing	1 (2.4)	2 (8.0)	2 (1.7)	5 (2.7)
Stage IV, *n* (%)				
At initial diagnosis/current stage	6 (14.6)/40 (97.6)	2 (8.0)/23 (92.0)	19 (16.2)/110 (94.0)	27 (14.8)/173 (94.5)
Elevated LDH (U/L) at baseline, *n* (%)	16 (39.0)	8 (32.0)	45 (38.5)	69 (37.7)
BRAF mutation status, *n* (%)				
None (no mutation detected)	0	0	3 (2.6)	
V600E/V600K	34 (82.9)/5 (12.2)	19 (76.0)/1 (4.0)	0/0	–
Unknown/other/missing data	1[b] (2.4)/1[c] (2.4)/0	2[b] (8.0)/0 /3[a] (12.0)	0/0/114 (97.4)	
NRAS mutation status, *n* (%)				
None (no mutation detected)	0	5 (20.0)	4[d] (3.4)	
Q61	0	0	100 (85.5)	
G12/13	0	0	2 (1.7)	
Unknown[e]/Missing data	1 (2.4)/40 (97.6)	0/20 (80.0)	1[f] (0.9)/10[g] (8.5)	
Prior antineoplastic medication, *n* (%)				
Any prior therapy	27 (65.9)	17 (68.0)	84 (71.8)	128 (69.9)
BRAF inhibitor	7 (17.1)	14 (56.0)	0	21 (11.5)
Number of regimens				
1	10 (24.4)	3 (12.0)	47 (40.2)	60 (32.8)
2	11 (26.8)	8 (32.0)	18 (15.4)	37 (20.2)
3	4 (9.8)	2 (8.0)	10 (8.5)	16 (8.7)
4	1 (2.4)	2 (8.0)	4 (3.4)	7 (3.8)
> 4	1 (2.4)	2 (8.0)	5 (4.3)	8 (4.4)
Chemotherapy	16 (39.0)	10 (40.0)	59 (50.4)	85 (46.4)
Dacarbazine	10 (24.4)	4 (16.0)	41 (35.0)	55 (30.1)
Immunotherapy	19 (46.3)	11 (44.0)	52 (44.4)	82 (44.8)
Interferon	10 (24.4)	7 (28.0)	19 (16.2)	36 (19.7)
Ipilimumab	6 (14.6)	6 (24.0)	28 (23.9)	40 (21.9)
Other	3 (7.3)	1 (4.0)	6 (5.1)	10 (5.5)

[a]Of these 4 patients, 3 had mucosal melanoma, and 1 had an unknown primary site of melanoma;
[b]V600E according to local laboratory;
[c]V600R according to local laboratory;
[d]Three Q61R (1 result received after database lock) and one G12 (result received after database lock);
[e]Any other mutation not known; known mutation includes all Q61, A59T, A11T, G12V, G13R;
[f]Q61R according to local laboratory;
[g]Three Q61R, two Q61L, two Q61K, two Q61, and one G12 mutation according to local laboratory.
LDH, lactate dehydrogenase; U/L, upper limit; WHO, World Health Organization.

Supplementary Table 2: Non-synonymous single nucleotide variant mutations in patients with BRAF or NRAS mutation with categorization based on findings in COSMIC

Patients with BRAF mutation in COSMIC											
**COSMIC**	**CDKN2A**	**PTEN**	**TP53**	**EZH2**	**TRRAP**	**EGFR**	**MYST3**	**AKT3**	**ARID1A**	**CTNNB1**	**GNAS**
**Pathogenic**	238C>T	112C>T	1024C>T	1936T>A	2165C>T	994C>T	80C>T	152T>G	4910G>A	133T>C	679C>G
	341C>T	675T>G	451C>T		7603G>A			49G>A	3440C>T	110C>A	
	205G>T	464A>G	794T>C		1577C>T				5965C>T	134C>A	
**Neutral**	71G>C										
**Unknown**									865C>T		
**Not in COSMIC**	151-1G>A				2165C>T	1574C>T	5791A>G		3619C>T		1268C>A
					11135C>T		4118C>T				241G>A
					9233C>A						736C>T
					2165C>T						558G>C
					3807G>C						622C>T
											1203G>A
											1736G>C
											422C>T

Patients with NRAS mutation in COSMIC							
**COSMIC**	**CDKN2A**	**TP53**	**CTNNB1**	**EP300**	**FBXW7**	**CD79B**	**EPHA3**
**Pathogenic**	220G>T	380C>T	94G>A	2773C>A	1787C>T	587A>G	2005G>A
	389T>G	454C>T	110C>G		1436G>A		1843G>A
	262G>T	541C>T	98C>G		1177C>T		1732G>A
	341C>T	637C>T	134C>A		1673C>T		2563C>T
	148C>T	661G>T	137T>C				2416G>A
	248A>C	722C>T	104T>C				
	238C>T	734G>C					
		742C>T					
		772G>A					
		800G>A					
		949C>T					
		986C>T					
		1009C>T					
**Neutral**	172C>T						
	44G>A						
**Unknown**							855G>A
**Not in COSMIC**		1036G>C	1693C>T	3673A>G	1844A>C		465G>A
			332C>A	2399C>T			621G>T
							2398A>G
							983C>T
							1969G>C

COSMIC; Catalogue of Somatic Mutations in Cancer.
Available online at: https://cancer.sanger.ac.uk/cosmic/gene/anlaysis.

Supplementary Table 3: Frequency of genetic pathway alterations in patients with *BRAF* or *NRAS* mutations

Patients with *BRAF* mutation		Patients with *NRAS* mutation	
**Pathway/process (associated genes)**	**Patients with alteration, *n* (%)**	**Pathway/process (associated genes)**	**Patients with alteration, *n* (%)**
All patients with analyzed samples	48	All patients with analyzed samples	78
Cell cycle pathway (*AURKA, CCND1, CCND3, CCNE1, CDK4, CDK6, CDKN2A, CDKN2C, RB1*)	33 (69)	Cell cycle pathway (*AURKA, CCND1, CCND3, CCNE1, CDK4, CDK6, CDKN2A, CDKN2C, RB1*)	45 (58)
PI3K (*AKT3, PIK3CA, PTEN*)	13 (27)	P53 (*MDM2, MDM4, TP53)*	21 (27)
Epigenetics (*ATRX, DNMT3A, EP300, MEN1, SMARCA4, SMARCB1, TET2, TRAAP*)	13 (27)	DNA damage response (*ATM, BRCA2, BRIP1, FANCA, FANCE, NBN, PALB2, PRKDC, RAD50*)	21 (27)
Transcription factors (*CEBPA, MED12, MITF, PAX5, RUNX1, RUNX1T1, SOX10, WT1*)	10 (21)	Transcription factors (*CEBPA, MED12, MITF, PAX5, RUNX1, RUNX1T1, SOX10, WT1*)	15 (19)
